# Re-irradiation of recurrent glioblastoma using helical TomoTherapy with simultaneous integrated boost: preliminary considerations of treatment efficacy

**DOI:** 10.1038/s41598-020-75671-9

**Published:** 2020-11-09

**Authors:** Donatella Arpa, Elisabetta Parisi, Giulia Ghigi, Alessandro Savini, Sarah Pia Colangione, Luca Tontini, Martina Pieri, Flavia Foca, Rolando Polico, Anna Tesei, Anna Sarnelli, Antonino Romeo

**Affiliations:** 1 Radiotherapy Unit, Istituto Scientifico Romagnolo per lo Studio e la Cura dei Tumori (IRST) IRCCS, Via P. Maroncelli 40, 47014 Meldola, Italy; 2 Medical Physics Unit, Istituto Scientifico Romagnolo per lo Studio e la Cura dei Tumori (IRST) IRCCS, Meldola, Italy; 3 Unit of Biostatistics and Clinical Trials, Istituto Scientifico Romagnolo per lo Studio e la Cura dei Tumori (IRST) IRCCS, Meldola, Italy; 4 Biosciences Laboratory, Istituto Scientifico Romagnolo per lo Studio e la Cura dei Tumori (IRST) IRCCS, Meldola, Italy

**Keywords:** Oncology, Cancer

## Abstract

Although there is still no standard treatment for recurrent glioblastoma multiforme (rGBM), re-irradiation could be a therapeutic option. We retrospectively evaluated the efficacy and safety of re-irradiation using helical TomoTherapy (HT) with a simultaneous integrated boost (SIB) technique in patients with rGBM. 24 patients with rGBM underwent HT-SIB. A total dose of 20 Gy was prescribed to the Flair (fluid-attenuated inversion recovery) planning tumor volume (PTV) and 25 Gy to the PTV-boost (T1 MRI contrast enhanced area) in 5 daily fractions to the isodose of 67% (maximum dose within the PTV-boost was 37.5 Gy). Toxicity was evaluated by converting the 3D-dose distribution to the equivalent dose in 2 Gy fractions on a voxel-by-voxel basis. Median follow-up after re-irradiation was 27.8 months (range 1.6–88.5 months). Median progression-free survival (PFS) was 4 months (95% CI 2.0–7.9 months), while 6-month PFS was 41.7% (95% CI 22.2–60.1 months). Median overall survival following re-irradiation was 10.7 months (95% CI 7.4–16.1 months). There were no cases of re-operation due to early or late toxicity. Our preliminary results suggest that helical TomoTherapy with the proposed SIB technique is a safe and feasible treatment option for patients with rGBM, including those large disease volumes, reducing toxicity.

## Introduction

Glioblastoma (GBM) relapses up to 90% of cases in close proximity to the site of resection or the initially irradiated tumor^1^. Median survival after progression is around 6 months^2^. The management of recurrent glioblastoma (rGBM) is challenging and there is still no standard treatment^3,4^. Radiotherapy (RT) could represent a valid treatment option. Historically, radiation oncologists have been cautious about re-irradiating brain tumors because of the risk of toxicity^5^. There are over 50 clinical studies in the literature on the re-irradiation of gliomas^6,7^, the majority retrospective in nature and reporting a variety of techniques, including brachytherapy, single-fraction stereotactic radiosurgery (SRS), fractionated stereotactic RT (FSRT) or hypofractionated stereotactic radiotherapy (HSRT)^3,8–18^.

Traditionally, the definition of the target volume for re-irradiation of rGBM is based on T1-weighted magnetic resonance imaging (MRI) with gadolinium^19^. Contrast enhancement is a consequence of the disruption of the blood–brain barrier, which does not necessarily reflect the real tumor extension in gliomas. Gross tumor mass has been detected beyond the margins of contrast enhancement, in the surrounding edema and even in adjacent normal-appearing brain tissue^20–22^. This prompted us to investigate the potential for re-irradiating rGBM using image-guided helical TomoTherapy (HT) with a simultaneous integrated boost (SIB) technique (HT-SIB) to simultaneously treat the tumor and the peritumoral area where there is suspected tumor diffusion. HT is capable of exploiting the biological advantages of SIB, an accelerated form of radiotherapy in which a higher dose can be delivered to the gross tumor volume (GTV), while a lower dose is simultaneously delivered to areas of subclinical disease^23–25^. Other studies have evaluated the feasibility of HT-SIB in the treatment of brain tumor and brain metastases, with good results^26–28^. HT combines fan-beam intensity-modulated radiotherapy with megavoltage computed tomography (MVCT) imaging for accurate patient positioning. Such a combination is a potentially viable alternative to conventional stereotactic frame systems for precision radiotherapy^28^. The purpose of the present retrospective study was to evaluate the efficacy and safety of this treatment in rGBM.

## Patients and methods

### Patient eligibility

This retrospective analysis included patients with rGBM treated in a single institute. Data on patient characteristics and treatment were retrospectively collected from computerized medical records in accordance with institutional ethical policies. Adult patients (aged ≥ 18 years) with clinical and /or imaging evidence of malignant GBM progression or recurrence as defined by RANO (response assessment for neuro-oncology) criteria^29^ received salvage re-irradiation (reRT). Main inclusion criteria are shown in Table 1. Exclusion criteria were as follows: (a) radiotherapy < 12 weeks prior to the diagnosis of progression if the lesion was in the radiation field and (b) T1-enhanced lesion and T2/Flair (T2-weighted fluid-attenuated inversion recovery) non-enhanced areas involving the brainstem and/or optic apparatus. At the time of recurrence, patients were evaluated for salvage treatment (including surgery, RT or chemotherapy) based on their clinical conditions, tumor site and volume, and hematologic rescue. A multidisciplinary team including a medical oncologist, radiation oncologist and neurosurgeon evaluated each clinical case to define the appropriate therapeutic strategy.

**Table 1:** Eligibility criteria.

Male or female, aged > 18 years
Karnofsky performance status (KPS) ≥ 60
Imaging confirmation of first tumor progression or re-growth, as defined by RANO criteria, at least 12 weeks after completion of radiotherapy, unless recurrence was outside the radiation field
Radiotherapy > 12 weeks prior to the diagnosis of recurrence or progression if the lesion was within the radiation field
Life expectancy > 12 weeks
If female and of child-bearing age, the patient was required to have a negative pregnancy test a maximum of 7 days before start of treatment

*RANO* response assessment in neuro-oncology.

### Imaging and delineation

**Figure 1:**
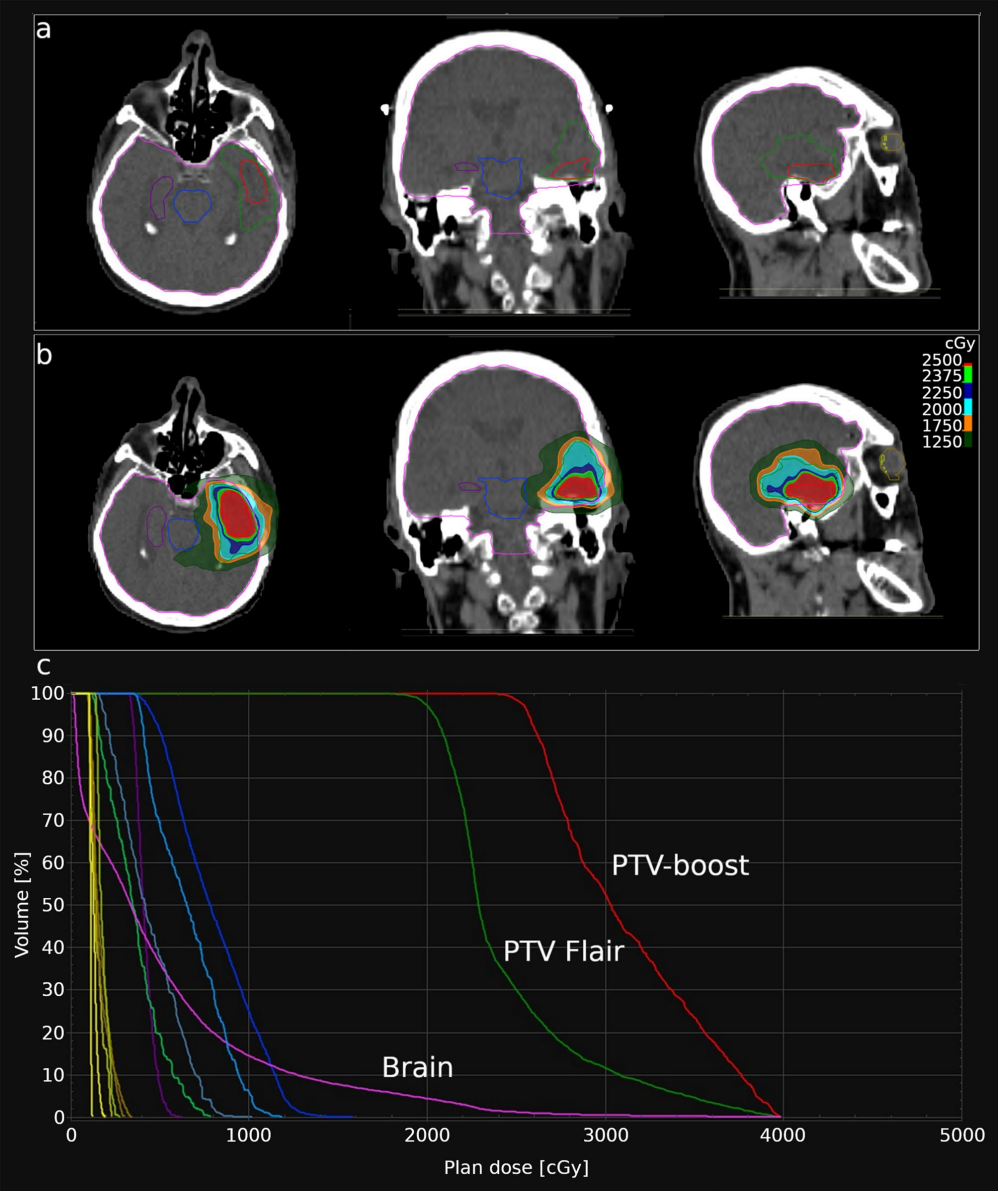
(**a**) Example of contouring of planning treatment volume (PTV)-boost (red line) and PTV Flair (green line). (**b**) Example of the dose distribution in the same patient. (**c**) Example of typical dose volume histograms (DVH).

Planning computed tomography (CT) (BrillianceBig Bore CT Philips, Crowley, UK) was obtained with a 1–3-mm slice thickness. Patients were placed in the supine position with arms close to their body and were immobilized with a frameless thermoplastic mask. A co-registration of volumetric CT and MR sequences (1–3-mm slice thickness T1, enhanced T1, Flair and T2 MRI) was performed was used to define the target and organs at risk (OAR). The planning target volume (PTV Flair) was defined on the area of peritumoral edema using T1, T2 and Flair sequences with a 2-mm margin expansion. The PTV-boost was defined as the visible tumor on enhanced T1-MRI with a 1-mm margin expansion (Fig. 1a). OARs were identified as healthy brain, optic chiasm, optic nerves and brainstem.

### Treatment planning

A total dose of 20 Gy was prescribed to the PTV Flair (99% isodose line covering 99% of the PTV), 25 Gy was prescribed to the PTV-boost in 5 daily fractions at the isodose of 67% (i.e. maximum dose within the PTV-boost was 37.5 Gy) (Fig. 1b). Planning was performed using Hi-art Helical TomoTherapy inverse planning software (TomoTherapy Inc., Madison, WI, USA).We evaluated the D98%^30^ (dose to 98% of the PTV = near-minimum dose), D2% (dose to 2% of the PTV = near-maximum dose) and D50% (dose to 50% of the PTV = median absorbed dose) for PTV Flair and PTV-boost.

Treatment toxicity was evaluated by converting the 3D-dose distribution to the equivalent dose in 2 Gy fractions (EQD2) on a voxel-by-voxel basis. EQD2 was defined as the total dose delivered in 2-Gy fractions at an alpha/beta (α/β) ratio of 2 Gy for normal brain tissue, optic pathway and brainstem due to the low repair capacity of these OARs^31^.

### Assessment of response and toxicity

The assessment of radiological and clinical response was based on MRI sequences obtained before and after HT-SIB. Baseline evaluation included volumetric T1 gadolinium-enhanced, fluid-attenuated inversion recovery imaging (FLAIR), axial T2-weighted imaging and DWI (diffusion-weighted imaging) MRI. We also used dynamic susceptibility contrast-enhanced (DSC) to establish progression/pseudoprogression and radiation necrosis. In difficult cases we used O-(2-[^18^F]fluoroethyl-)-l-tyrosine (^18^F-FET) PET/CT to support the differential diagnosis of PD or treatment-related changes. The assessment of toxicity was based on clinical examination carried out on the first, third and last day of re-irradiation, then 40 days after the end of re-RT and every 3 months thereafter. Patients were followed until disease progression or death. All toxicities were recorded and graded according to NCI CTCAE (National Cancer Institute Common Toxicity Criteria for Adverse Events), version 4.3.

### Statistical analysis

Overall survival (OS) from primary diagnosis was defined as the time from surgery until death from any cause, or until the date of the last follow-up. Progression-free survival (PFS) after salvage therapy was defined as the time from the start of salvage TomoTherapy until disease progression or last follow-up. OS after salvage therapy was defined as the time from the start of salvage tomotherapy until death from any cause or until the date of the last follow-up. Time from the initial RT to salvage RT was defined as the time from date of adjuvant RT until the date of salvage TomoTherapy.

Event-time distributions were estimated using the Kaplan–Meier method, with 95% confidence intervals (95% CI) provided where appropriate. The role of the stratification factor was analyzed with the log-rank test. Median and range were calculated for continuous variables, while numbers and percentages were used for categorical variables. Statistical analyses were carried out with STATA/MP 15.1 for Windows (StataCorp LLC, College Station, TX, US).

### Ethics approval and consent to participate

This study was approved by the Ethics Committee IRST IRCCS AVR (approval number L2P1912 of 15/05/2019) and carried out in accordance with the ethical standards laid down in the 1964 Declaration of Helsinki. In accordance with Italian legislation, written informed consent for observational retrospective studies carried out in Scientific Institutes for Research, Hospitalization and Healthcare (IRCCS) was not required.

## Results

### Patients

Twenty-four consecutive patients (10 females and 14 males) with rGBM were treated at our institute between August 2008 and December 2017. Median age was 57 years (range 27–70 years). All patients had a Karnofsky Performance status (KPS) of ≥ 60. The entire cohort underwent initial post-operative fractionated RT with a standard dose schedule of 60 Gy in 30 fractions. Specific patient characteristics are reported in Table 2 and Supplementary Table S1. The median interval between primary RT and salvage RT was 18.7 months (range 3.6–64.8 months). Ten (41.66%) patients underwent HT-SIB as first salvage treatment at relapse, 14 (58.33%) as second treatment after salvage chemotherapy (5 patients) or salvage surgery (9 patients). These last 9 patients progressed and were re-irradiated a median of 4.7 months after re-surgery (95% CI 2.1–19.0).

**Table 2:** Patient characteristics.

Median age at primary (range)]	No. (%)
**Gender**	
Male	14 (58.33)
Female	10 (41.67)
KPS ≥ 60	24
**Histology**	
Glioblastoma	24
**Tumor site**	
Frontal lobe	8 (33.35)
Temporal lobe	6 (25.0)
Parietal lobe	5 (20.83)
Insula	2 (8.33)
Callous body	2 (8.33)
Occipital lobe	1 (4.16)
**Post-operative radiotherapy (n patients)**	
2 Gy daily (total dose 60 Gy)	24
Median interval between post-operative radiotherapy and salvage HT-SIB, months [range]	18.7 [3.6–64.8]
**Salvage therapy before re-irradiation**	
No	10 (41.67)
Re-surgery	9 (37.5)
Chemotherapy or immunotherapy	5 (20.83)

*HT-SIB* helical TomoTherapy-simultaneous integrated boost.

### Treatment characteristics

The median volume of PTV Flair was 107 cc (range 9.8–395.0 cc) and the median PTV-boost was 33 cc (range 6.7–196.4 cc). In patients who were borderline for re-irradiation, especially those with large tumor volumes, the final decision to re-irradiate was based on the following: KPS of the patient, age at time of HT-SIB, interval between the first and second radiotherapy course, and the proximity of critical organs to the targets. The median values of D98%, D2% and D50% for the PTV Flair and PTV- boost are shown in Table 3. We tailored the treatment for each patient, analyzing case by case. The dose to the OARs and the EQD2 are reported in Table 4. We considered a cumulative EQD2 Gy_2_ value of < 55 Gy_2_ for optic nerves and chiasm and < 60 Gy_2_ for brainstem as dose constraints for planning. No dose constraint violations were registered.

**Table 3:** Treatment details of HT-SIB re-irradiation.

	Mean	Maximum	Minimum
PTV Flair volume cc	107 cc	395 cc	9.8 cc
D98%	1970 cGy	2477 cGy	1866 cGy
D2%	3402 cGy	3789 cGy	2270 cGy
D50%	2256 cGy	2655 cGy	2025 cGy
PTV-boost volume cc	33 cc	196.4 cc	6.7 cc
D98%	2470 cGy	3504 cGy	2270 cGy
D2%	3715 cGy	4008 cGy	3160 cGy
D50%	3021 cGy	3098 cGy	2853 cGy

*HT-SIB* helical TomoTherapy-simultaneous integrated boost, *D* dose, *cc* cubic centimeter, *cGy* centigray.

**Table 4:** Organ-at-risk doses.

	Median maximum dose, Gy (range)	EQD2 Dmax, Gy_2_ (range)
Right optic nerve	3.26 (0.24–18.96)	2.76 (0.12–27.45)
Left optic nerve	2.99 (0.29–17.4)	2.13 (0.14–23.94)
Chiasm	9.28 (0.37–23.31)	6.29 (0.41–31.39)
Brainstem	7.59 (0.73–22.35)	7.01 (0.50–32.45)
Healthy brain	36.69 (26.19–41.74)	53.02 (40.57–68.75)

*EQD2* total dose delivered in 2-Gy fractions at alpha/beta (α/β) ratio of 2 Gy for normal brain tissue, optic pathway, brainstem; *Dmax* maximum dose.

### Outcomes

**Figure 2:**
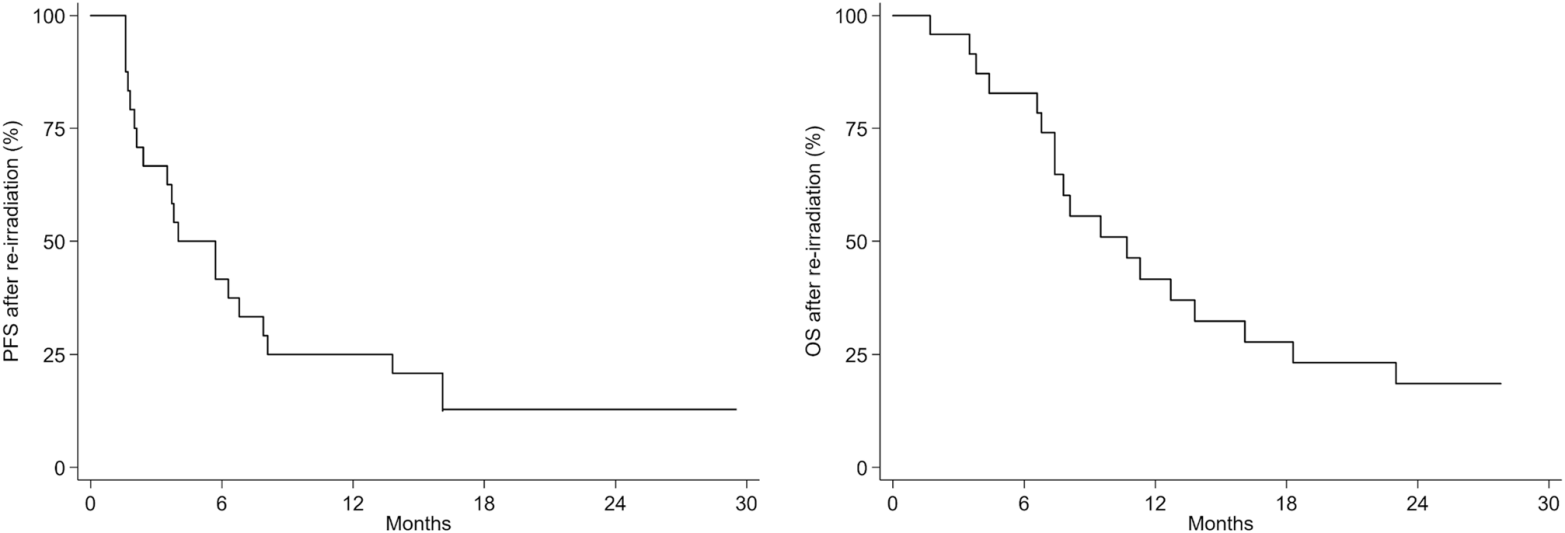
Progression-free survival (PFS) and overall survival (OS) after helical TomoTherapy with simultaneous integrated boost.

Median follow-up from re-irradiation was 27.8 months (range 1.6–88.5 months). During treatment dexamethasone ≥ 2 mg was administered to all patients. No acute or late neurologic toxicity > grade 2 (CTCAE version 4)^32^ was observed. All 24 patients completed the prescribed radiation dose without interruption. All but 3 required daily doses of dexamethasone ≥ 4 mg for > 8 weeks after HT-SIB. Median PFS (mPFS) for all patients was 4 months (95% CI 2.0–7.9 months), 3-month PFS was 66.7% (95% CI 44.3–81.7 months) and 6-month PFS was 41.7% (95% CI 22.2–60.1 months). Median OS (mOS) of re-irradiated patients was 10.7 months (95% CI 7.4–16.1 months) (Fig. 2). No significant differences were observed on the basis of age at the time of HT-SIB or on the basis of re-surgery before re-RT. Patients aged < 56 years had a mOS of 7.8 months (95% CI 4.4–18.3) vs*.* 10.7 months (95% CI 3.5–23.0) for those ≥ 56 years (p = 0.881). Patients who underwent re-surgery before re-irradiation showed a mOS of 10.7 months (95% CI 4.4–23.0) vs*.* 8.1 months (95% CI 3.8–18.3) for those who did not require re-surgery (p = 0.621).

At the time of the last observation, 6 (25%) patients were still alive, while 18 (75%) had died. Among the former group, 4 patients progressed according to RANO criteria after a median of 4 months (range 4–5 months), while 2 had a complete response (CR) to HT-SIB lasting 24 months and 27 months. The latter patient relapsed (2.5-cm lesion) within 2 cm of the re-irradiated lesion and was able to undergo second surgery because of good performance status (KPS 90%), the small size of the new lesion and the long DFS. Histology showed methylated MGMT GBM (34%), negative IDH1 and IDH2, Ki-67 18%, and no radionecrosis. mOS for the entire group calculated from the primary diagnosis was 44.4 months (95% CI 25.1–50.4 months).

## Discussion

Several authors have investigated the feasibility of the SIB technique for the treatment of primary and metastatic brain lesions^23,24,26,28,33–38^. Rodrigues et al.^24^ delivered 60 Gy in 10 fractions to 1–3 brain metastases synchronously with 30 Gy whole brain irradiation (WBRT) using HT. The doses were delivered without dose-limiting central nervous system toxicity, as assessed 3 months after treatment. Bauman et al.^28^ confirmed the feasibility of SIB for individual brain metastases during a course of WBRT using HT. All of the studies confirmed that HT-SIB is capable of delivering a homogeneous brain dose and reasonably conformal dose to metastases, with surgical precision. Baisden et al.^36^ treated primary brain tumors with the HT-SIB technique, with a significant sparing of normal brain parenchyma compared to conventional HT with sequential boost. A dose of 50 Gy was prescribed to the larger PTV1, while the PTV-boost received a total of 60 Gy. The authors reported that HT-SIB plans resulted in a reduction in the mean brain dose, the volume of normal brain receiving 45 Gy (V45) and the volume of normal brain receiving 5 Gy compared with sequential boost plans. HT proved capable of treating multiple targets in the same region simultaneously at varying dose levels, lending itself naturally to SIB methods. The main advantages in using this technique to treat rGBM are (1) reduction in treatment time, with subsequent decrease in patient discomfort; (2) delivery of a higher biologically equivalent dose to the PTV-boost, which may improve tumor control; (3) delivery of 2 dose levels to PTVs without increasing brain toxicity; (4) possibility of treating large tumor volumes, with a low risk of acute and sub-acute toxicity.

In the present study the HT-SIB plans were created to deliver a total dose of 20 Gy to the PTV Flair (99% isodose line covering 99% of the PTV) and 25 Gy to the PTV-boost in 5 fractions. The prescribed dose isoline to the PTV-boost was 67% (i.e. maximum dose within the PTV-boost was 37.5 Gy). By using a 67% isodose prescription, the PTV-boost received a much higher and inhomogeneous dose than that of the PTV Flair (Fig. 1c). The inhomogeneous dose inside the PTV-boost, in delivering a higher dose to the hypoxic tumor cells, theoretically results in improved local control. Recently, Lucia et al.^39^ established that stereotactic radiotherapy delivery with inhomogeneous dose distribution led to better local control than homogeneous distribution in brain metastases, especially higher D2% values. In fact, our D2% values of the PTV-boost ranged from 3160 to 4008 cGy, while those of the PTV Flair ranged from 2270 to 3789 cGy. The ICRU (International Commission on Radiation Units) previously recommended that dose values in the PTV be confined within the range of 95–107% of the prescribed dose^40^. These constraints are not used in stereotactic radiotherapy (SRT) as some clinicians prefer to deliver a high dose to the middle of the target. However, as highlighted in the ICRU Report 91^41^ on Stereotactic Radiotherapy, the dose is often prescribed to the 60–80% isodose (relative to maximum dose) line which is located on the outline of the PTV.

In the re-irradiation setting^42,43^, a smaller irradiated volume is preferable in terms of toxicity, while limiting treatment to contrast-enhancing lesions may lead to lower local control given the invasiveness of gliomas^14^. Accurately determining tumor spread is especially difficult in diffuse tumors, thus increasing the risk of local failure. Contouring recurrent high-grade glioma on the basis of gadolinium-enhanced T1-weighted gadolinium MRI alone guarantees a specificity of only 50%^44^. Other imaging modalities have been used to delineate the GTV, including MR-spectroscopy, perfusion-weighted imaging and diffusion weighted imaging^10,45^, 11C-methionine positron emission tomography (MET-PET)^46^, and18 F-dihydroxyphenylalanine (DOPA) PET^47^. In the majority of studies^12,17,48–50^, the authors delineate only contrast-enhancing lesion on T1-weighted images GTV, while the clinical target volume (CTV) is equal to the GTV. A further millimetric margin is usually added to the CTV to create a planning target volume (PTV). As reported by some authors^11,15,51^, we also included the area of peritumoral edema, defined on the Flair sequences with a 2-mm margin expansion, in the PTV Flair, whereas the PTV-boost was defined as the visible tumor on enhanced T1-MRI with a 1-mm margin expansion.

The median PTV Flair value was 107 cc (range 9.8–395 cc) while that of the PTV-boost was 33 cc (range 6.7 cc to 196.4 cc). Despite the volume of treatment, mPFS was 4 months (95% CI 2.0–7.9 months), 3-month PFS was 66.7% (95% CI 44.3–81.7 months) and 6-month PFS was 41.7% (95% CI 22.2–60.1 months). mOS was 10.7 months (95% CI 7.4–16.1 months). In the literature, studies on the re-irradiation of rGBM rarely report the mPFS, while mOS ranges from 6.7 to 12 months^10–18^. Diverse radiotherapy regimens are used, doses ranging from 22 to 35 Gy in 5–10 sessions, with treatment volumes of 24 cm^3^ to 69.5cm^3^. Vodemark et al.^17^ treated recurrent high grade glioma (median volume 15 ml) with a median dose of 30 Gy in 6 fractions of 5 Gy/die, reporting a mPFS of 4.6 months and a mOS of 7.9 months but no severe toxicity. Fokas et al.^48^ re-irradiated 53 rGBM patients (median volume of 35 ml) with HSRT, delivering a median total dose of 30 Gy in a median of 10 fractions. Twelve-month actuarial PFS was 22%, with a mOS of 9 months. Re-irradiation was well tolerated (no acute or late toxicity > grade 2). Ernst-Stecken et al.^52^ evaluated the efficacy and side-effects of HSRT of 35 Gy in 5 fractions (3 times/week), median volume 22.4 ml (0.77–21.94 ml), observing no severe toxicity and a 12-month PFS of 53%. Toxicity reported in the literature is highly variable, some authors observing no severe toxicity, others reporting 12.5% of pathologically proven radionecrosis^15^. In our series there were no cases of acute neurologic toxicity > grade 2 (CTCAE vers. 4.03) during treatment and no re-operations were needed due to early or late toxicity.

Mayer et al.^53,54^ published a review on radiation tolerance of the human brain, concluding that radiation-induced necrosis of normal brain tissue occurred with a cumulative equivalent dose of 2 Gy fractions > 100 Gy_2._ Smaller volumes and more conformal techniques such as FSRT and SRS allow safe delivery of higher EQD2 cumulative doses (90–133.9 Gy and 11.6–137.2 Gy, respectively).The authors hypothesized that the re-irradiation and EQD2 cumulative doses increased when techniques such as FSRT and SRS were used, without, however, increasing the risk of normal brain necrosis. In a review on cranial re-irradiation, Nieder et al.^6^ reported that a fraction size of 3–5 Gy was well tolerated in limited-volume recurrences (< 75 ml) as long as the total dose was limited to 30 Gy-35 Gy.

In our study, the EQD2 value of 25 Gy to the isodose line of 67% was 87.58 Gy_2_, while the EQD2 of 20 Gy was 74.8 Gy_2,_ based on the median value of D2% (37.15 Gy and 34.02 Gy, respectively).Taking into account the uniformity of the initial radiation treatment in which the healthy brain received a uniform dose of 60 Gy (2 Gy/die for 30 sessions) and the EQD2 calculated for both prescription doses, we determined a cumulative EQD2 of 147.58 Gy_2_ and 134.8 Gy_2_. Such high values carry the risk of severe toxicity, which, on the contrary, we did not observe. HT-SIB probably delivers a marginal low dose that inhomogeneously increases inside the target. It enables large tumor volumes to be treated that previously would not have been contemplated due to the risk of toxicity. The HT-SIB technique, in creating highly conformal dose distribution, potentially reduces the dose to surrounding critical structures (Table 4). The data for our case series are modest, with mOS percentages similar to those of other re-irradiation studies. It is clear, however, that greater efforts are needed to improve the prognosis of patients with recurrent glioblastoma.

The limitations of this study include its retrospective nature, selection bias, lack of biological information and various treatment factors, including chemotherapy and surgery before re-irradiation, and evaluation of quality of life, all of which made it difficult to interpret outcome.

## Conclusions

The preliminary results from the present study suggest that HT with the proposed SIB technique is a safe and feasible, albeit not curative, treatment option for patients with rGBM, including those with large tumors. Further studies in primary and recurrent settings are needed to confirm our findings.

## Supplementary information

Supplementary Table S1.

## Data Availability

The datasets used and/or analyzed during the current study are available from the corresponding author on reasonable request.

## Abbreviations

CT: Computed tomography
CTV: Clinical target volume
EQD2: Equivalent dose in 2 Gy fractions
FSRT: Fractionated stereotactic radiotherapy
GBM: Glioblastoma
GTV: Gross tumor volume
HSRT: Hypofractionated stereotactic radiotherapy
HT: Helical tomotherapy
HT-SIB: Helical tomotherapy with a simultaneous integrated boost technique
MRI: Magnetic resonance imaging
MVCT: Megavoltage computed tomography
OAR: Organs at risk
OS: Overall survival
PFS: Progression-free survival
PTV: Planning target volume
reRT: Re-irradiation
rGBM: Recurrent glioblastoma multiforme
RT: Radiotherapy
SIB: Simultaneous integrated boost
SRS: Stereotactic radiosurgery
WBRT: Whole brain irradiation
