# Supplementary material for: Re-irradiation of recurrent glioblastoma using helical TomoTherapy with simultaneous integrated boost: preliminary considerations of treatment efficacy
Source: Sci Rep. 2020 Nov 9;10:19321. doi: 10.1038/s41598-020-75671-9 (PMC7653937; doi:10.1038/s41598-020-75671-9)
Supplement: Supplementary file 1 — Supplementary Table S1. [file 41598_2020_75671_MOESM1_ESM.pdf]

RESEARCH ARTICLE

Re-irradiation of recurrent glioblastoma using helical TomoTherapy with simultaneous integrated boost: preliminary considerations of treatment efficacy

Donatella Arpa[1,*], Elisabetta Parisi[1], Giulia Ghigi[1], Alessandro Savini[2], Sarah Pia Colangione[1], Luca Tontini[1], Martina Pieri[1], Flavia Foca[3], Rolando Polico[1], Anna Tesei[4], Anna Sarnelli[2] & Antonino Romeo[1]

[1]Radiotherapy Unit, Istituto Scientifico Romagnolo per lo Studio e la Cura dei Tumori (IRST) IRCCS, Meldola, Italy. [2]Medical Physics Unit, Istituto Scientifico Romagnolo per lo Studio e la Cura dei Tumori (IRST) IRCCS, Meldola, Italy. [3]Unit of Biostatistics and Clinical Trials, Istituto Scientifico Romagnolo per lo Studio e la Cura dei Tumori (IRST) IRCCS, Meldola, Italy. [4] Biosciences Laboratory, Istituto Scientifico Romagnolo per lo Studio e la Cura dei Tumori (IRST) IRCCS, Meldola, Italy

Supplementary Table S1. Detailed patient characteristics

Surgery at initial diagnosis	MGMT methylation status	Adjuvant treatment	Adjuvant TMZ	Salvage treatment before HT-SIB	Interval between first RT and HT-SIB (months)	Therapy at PD after HT-SIB	PFS after HT-SIB (months)	OS after HT-SIB (months)	OS after initial diagnosis (months)
GTR	Unknown	RT/TMZ	TMZ	Re-surgery	17	Fotemustine	6	8	26
GTR	Unknown	RT/TMZ	-	Re-surgery	44	Unknown	20	88.5	135
GTR	Unmethylated	RT/TMZ	-	Re-surgery	10	BSC	2	7	27
STR	Unmethylated	RT/TMZ	TMZ	-	9	BSC	7	7	19
STR	Unmethylated	RT/TMZ	TMZ	-	4	Fotemustine	2	4	11
STR	Unknown	RT/TMZ	TMZ	-	29	BSC	1.6	1.6	32
STR	Methylated	RT/TMZ	TMZ	Re-surgery	33	Second Re-RT	6	13	47
GTR	Methylated	RT/TMZ	TMZ	-	13	BSC	4	4	20
BIOPSY	Methylated	RT/TMZ	TMZ	Fotemustine	34	Rechallenge TMZ	2	10	44
STR	Unmethylated	RT/TMZ	TMZ	Bevacizumab	9	BSC	2	11	23
STR	Methylated	RT/TMZ	TMZ	Re-surgery	13	BSC	2	4	19
STR	Unmethylated	RT/TMZ	TMZ	Re-surgery	31	Etoposide+ carboplatin	4	16	48
GTR	Unmethylated	RT/TMZ	TMZ	Re-Surgery	8	Fotemustine	6	7	18
GTR	Methylated	RT/TMZ	TMZ	Bevacizumab	35	BSC	2	4	25
GTR	Methylated	RT/TMZ	TMZ	-	60	Re-surgery followed by TMZ	24	36	98
GTR	Unmethylated	RT/TMZ	TMZ	Fotemustine	13	PCV	6	9	25
GTR	Methylated	RT/TMZ	TMZ	-	33	TMZ	8	11	47
GTR	Methylated	RT/TMZ	TMZ	-	11	Bevacizumab	4	7	22
GTR	Methylated	RT/TMZ	TMZ	-	9	PCV	4	28	40
GTR	Unknown	RT/TMZ	TMZ	-	65	Unknown	10	14	81
STR	Unmethylated	RT/TMZ	TMZ	Fotemustine	41	Bevacizumab	2	7	50
GTR	Methylated	RT/TMZ	TMZ	Re-surgery	46	Fotemustine	2	8	58
STR	Unknown	RT/TMZ	TMZ	Re-surgery	19	Bevacizumab	16	18	40
STR	Methylated	RT/TMZ	TMZ	-	21	Bevacizumab	4	27	51

GTR: gross total resection; STR: subtotal resection; TMZ: temozolomide; BSC: best supportive care; HT-SIB: helical TomoTherapy-simultaneous integrated boost; Re-RT: re-irradiation: PCV: procarbazine, lomustine and vincristine.
